# Morphometric Characterization and Zoometric Indices of High-Andean Creole Cows from Southern Peru

**DOI:** 10.3390/vetsci12080782

**Published:** 2025-08-20

**Authors:** Ruth Ccalta, Rito Felipe Huayta Arizaca, Elvis Lizandro Salcedo Quispe, Anthony Valverde, Hernán Carlos Cucho Dolmos, Ali William Canaza-Cayo, Alex Yony Acuña Leiva, Richard Estrada

**Affiliations:** 1 Instituto Nacional de Innovación Agraria, Estación Experimental Andenes Cusco, Cusco 08003, Peru; huaytaarizacaritofelipe@gmail.com (R.F.H.A.); salcedoelvis39@gmail.com (E.L.S.Q.); 2 Facultad de Agronomía y Zootecnia, Escuela Profesional de Zootecnia, Universidad Nacional San Antonio Abad del Cusco, Cusco 08006, Peru; hernan.cucho@unsaac.edu.pe; 3 Laboratorio de Reproducción Animal, Centro de Investigación y Desarrollo en Agricultura Sostenible del Trópico Húmedo, Escuela de Agronomía, Instituto Tecnológico de Costa Rica, Campus Tecnológico Local San Carlos, Alajuela 223-21002, Costa Rica; anvalverde@itcr.ac.cr; 4 Facultad de Ciencias Agrarias, Escuela Profesional de Ingeniería Agronómica, Universidad Nacional del Altiplano, Puno 21103, Peru; alicanaza@unap.edu.pe; 5 Departamento de Estadística, Instituto de Ciencias Exactas y Tecnológicas, Universidad Federal de Lavras, Código Postal 3037, Lavras CEP 37200-900, MG, Brazil; 6 Dirección de Desarrollo Tecnológico Agrario, Instituto Nacional de Innovación Agraria, La Molina, Lima 15024, Peru; alexacunaleiva6@gmail.com (A.Y.A.L.); richard.estrada.bioinfo@gmail.com (R.E.)

**Keywords:** Creole cattle, morphometry, zoometric indices, biotypification, high Andean livestock farming

## Abstract

High-Andean Creole cattle represent a genetic resource with significant adaptive value in high-altitude ecosystems. In this study, 151 Creole cows from the provinces of Canas and Quispicanchis (Cusco, Peru) were evaluated to characterize their morphometry, calculate zoometric indices, and establish functional biotypes. Ten morphometric parameters were measured, and eight structural indices were calculated. Four differentiated biotypes were identified through the multivariate analysis and hierarchical clustering of quantitative variables (BI, BII, BIII, and BIV). Morphological differences were statistically significant (*p* < 0.05) and associated with different productive orientations. Biotype I showed greater thoracic development and a compact structure, which is compatible with intermediate beefaptitude; Biotype II exhibited more elongated proportions and a lower weight, indicating a dairy tendency; Biotype III stood out for its muscle mass, thoracic depth, and pelvic width, evidencing a clear beef orientation; and Biotype IV combined a higher weight, rectangular conformation, and wide pelvis, showing potential for beef production and ease of calving. Functional differentiation was supported by Principal Coordinates Analysis (PCoA) and PERMANOVA tests. Correlations between morphometric variables and zoometric indices revealed anatomical patterns consistent with the productive functions proposed. The results highlight the morpho-functional diversity of Andean Creole cattle and their potential for conservation and improvement programs in extensive highland systems.

## Simple Summary

High-Andean Creole cattle is a valuable breed highly adapted to the ecosystems of the Southern Andes of Peru, 3500 m above sea level. Through body measurements and zoometric indices, four biotypes with differentiated morpho-functional characteristics were identified. Biotypes I, III, and IV showed greater muscular development and a wide pelvis, reflecting an aptitude for meat production and dual-purpose use (meat and milk). In contrast, Biotype II exhibited lighter proportions, associated with a clear dairy orientation. These results highlight the diversity of Andean Creole cattle and their potential to be incorporated into conservation and improvement programs in extensive high-altitude livestock systems, thereby contributing to the development and well-being of the highland population.

## 1. Introduction

During the conquest of the Americas, Spanish colonizers introduced cattle to Peru in the 16th century. Over time, these animals successfully adapted to the high-Andean environment. From a genetic perspective, Creole cattle in this region retain a high percentage of haplotypes inherited from their Bos taurus ancestry originating from the Iberian Peninsula, mainly represented by haplogroups Y1 (19%) and Y2 (81%) [1,2]. Recent studies have also identified genetic influences from African breeds [3]. This genetic diversity has facilitated the adaptation of Creole cattle to the harsh conditions of the Peruvian highlands, located 3500 m above sea level.

To this day, the rearing of Creole cattle has been essential for ensuring food security and economic stability, and it is deeply intertwined with the culture of the Andean populations in Southern Peru [4]. Their disease resistance, ability to survive with lower nutritional requirements, and reproductive capacity make them ideal for regions situated between 3500 and 4500 m in elevation, where climatic conditions are particularly challenging [5]. Recent genetic studies have provided valuable insights into Peruvian Creole cattle’s genetic diversity and population structure. For instance, in an analysis of 63 DNA samples from Creole cattle in the Arequipa region, a high genetic diversity was found, with 97.58% single-nucleotide polymorphisms (SNPs) and an expected heterozygosity of 0.41 ± 0.01, indicating significant genetic variability that contributes to their adaptability to the harsh conditions of the Peruvian Andes [6]. Moreover, the sequencing and assembly of the complete genome of Peruvian Creole cattle revealed 19,803 protein-coding genes and 40.22% repetitive DNA, with retroelements representing 32.39% of the total [7].

Despite their adaptive advantages, in the last fifteen years, Creole cattle have been progressively displaced in many areas by cosmopolitan breeds such as Brown Swiss, Holstein, Angus, and Simmental, which offer higher yields in meat, milk, and dual-purpose production [3]. This shift responds to the need to meet the demands of highland farmers, leading to the greater introduction of these breeds, often at the expense of local Creole populations. Nevertheless, it is crucial to acknowledge that Creole cattle continue to play unique and valuable roles in high-altitude production systems [8].

In Peru, several Creole cattle biotypes currently exist [6]. In the Amazon region, three distinct biotypes have been identified based on coat color and morphometric measurements [8]. In the Puno region, three biotypes have been classified according to coat type: black, red, and brindle [9]. In other Latin American countries, such as Ecuador, four Creole cattle biotypes have been reported [10,11]. However, in the Cusco region of Peru, no studies have yet been conducted to identify the biotypes present. Therefore, the present study aimed to analyze the morphometric characteristics and zoometric indices of high-Andean Creole cattle in Southern Peru.

## 2. Materials and Methods

### 2.1. Ethical Considerations and Study Location

**Figure 1:**
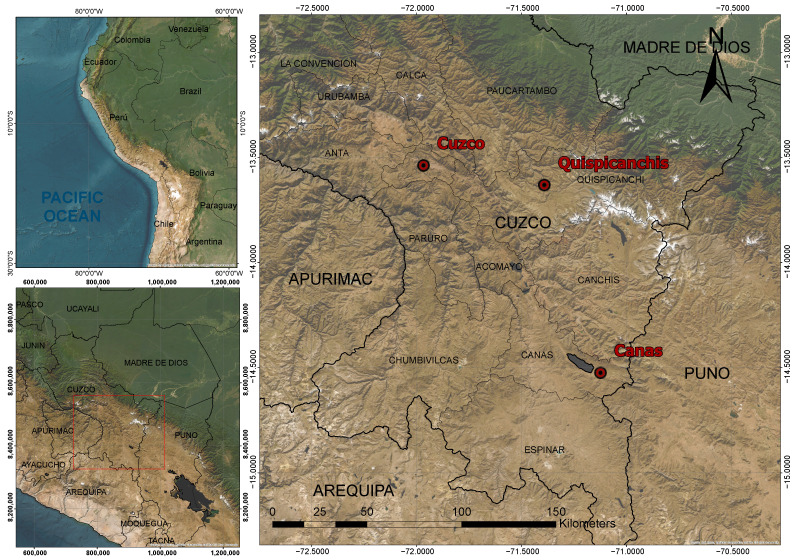
Study site: Cusco Region, Peru. (**Left**): Country: Peru, with the Cusco Region highlighted in red. (**Top right**) Quispicanchis Province (Ccarhuayo, Ocongate, and Marcapata districts). (**Bottom right**): Canas Province (Checca, Kunturkanki, and Layo districts).

This research was conducted following the legal framework established by the Animal Welfare Law No. 30407, applicable to all laboratories and public and private higher education institutions in Peru. The study was carried out in the districts of Kunturkanki, Layo, Checca, Ccarhuayo, Ocongate, and Marcapata, located in the provinces of Canas and Quispicanchis in the Cusco department, situated in the Southern Andean region of Peru (Figure 1). Evaluations were conducted at an average altitude of 4009 m above sea level (latitude: 13°72′01″ S; longitude: 71°40′07″ W).

### 2.2. Morphometric Measurements and Zoometric Indices

Morphometric measurements and zoometric indices were performed on Creole cows from the provinces of Canas and Quispicanchis. A total of 151 cows were used (Figure S1), distributed into three groups according to dental age: 29 cows aged 2.5–3 years, 49 cows aged 3.5–4 years, and 73 cows aged 4.5 years and older [12]. Measurements were taken between 7:00 and 10:00 a.m. A measuring stick was used for morphometric evaluations, and the following traits were measured: head length (HL), head width (HW), withers height (WH), thoracic perimeter (TP), body length (BL), thoracic depth (TD, rump length (RL), rump width (RW), cannon bone perimeter (CBP), rump height (RH), and body weight (BW). All measurements were expressed in centimeters [5]. These Creole cows are managed under an extensive production system, grazing on native high-Andean pastures. The cows were classified based on nine predominant coat color patterns: (A,B) black = 59; (C) reddish bay = 19; (D) dark brown = 20; (E) light brown = 13; (F) Black overo “callejón” = 9; (G) “Barrendo/Mora Negra” = 11; (H) smoky/Cardeno = 12; (I) brindle “Atigrado” = 6; and (Jmulatto = 2. The classification of coat color patterns was based on those described by Rojas-Espinoza et al. [9]. The body weight was measured with a bovinometric tape (Instruflex^®^, EOCH Peruana SRL, Lima, Perú).

Some indices were used to determine body conformation and functional aptitude, including the anamorphosis index (AI) calculated as the (thoracic perimeter)^2^ divided by the withers height and multiplied by 100; the body index (BI), calculated as the body length divided by the thoracic perimeter and multiplied by 100; the pelvic index (PI), calculated as the rump width divided by the rump length and multiplied by 100; the proportionality index (PRI) (also referred to as the lateral body ratio or relative shortness), calculated as the withers height divided by the body length and multiplied by 100; the cephalic index (CI), calculated as the head width divided by the head length and multiplied by 100. The dactyl-thoracic index (DTI), associated with dairy capacity, was calculated as the cannon bone perimeter divided by the thoracic perimeter and multiplied by 100. For beef aptitude, the pelvic-transversal (TI) index was calculated as the rump width divided by the withers height and multiplied by 100, and the pelvic-longitudinal index (LI) was calculated as the rump length divided by the withers height and multiplied by 100 [5].

### 2.3. Statistical Analysis

A total of 151 morphometric measurements from Creole cows were used for classification into biotypes through a dendrogram obtained via the hierarchical clustering of both quantitative traits. The dendrogram was constructed based on these analyses, applying a cutoff at 60% of the largest distance to define the clusters. Before this, all morphometric and zoometric indices were subjected to the Shapiro–Wilk test to assess the normality of residuals and to remove outliers. A one-way ANOVA was conducted to identify statistical significance. The analysis showed significant differences (*p* < 0.05) between the observed biotypes. Additionally, a Principal Coordinates Analysis (PCoA) was performed, complemented by a PERMANOVA test to validate the morphological differentiation among biotypes. Finally, Spearman correlation analyses were conducted to explore significant associations between morphometric variables and zoometric indices. The data were analyzed using the R statistical software package version 4.5.0.

## 3. Results

### 3.1. Biotypes and Morphometric Measurements

**Figure 2:**
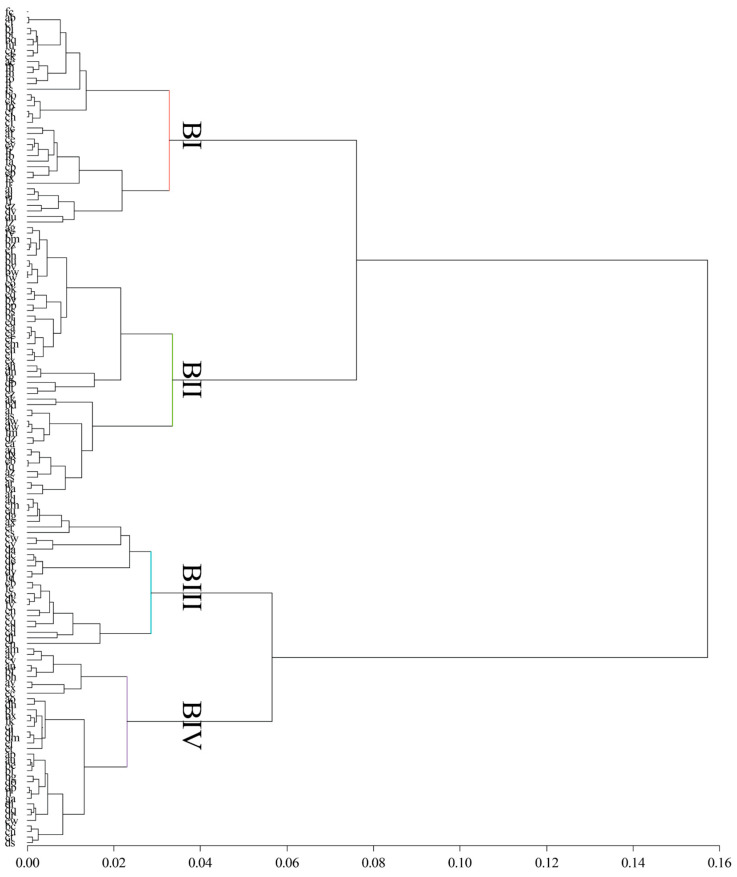
Dendrogram using the Ward method—R-scale cluster combination.

In the present study, four differentiated biotypes of Creole cows (Biotype I, Biotype II, Biotype III, and Biotype IV) were identified through hierarchical cluster analysis. The classification obtained was validated by an analysis of variance for hypothesis testing, which was highly significant (*p* < 0.001), confirming the existence of statistical differences among the defined biotypes (Figure 2).

The morphometric measurements of the Creole cows revealed significant differences among the four analyzed biotypes. Head width was greater in Biotype IV, while head length was significantly higher in Biotypes III and IV. The wither height showed higher values in Biotype III. The thoracic perimeter was greater in Biotypes BIII and BIV. Regarding body length, cows from Biotype BIII had the highest values, followed by Biotypes BII, BIV, and BI. Thoracic depth and rump length were greater in Biotype BIII. Rump width showed significant differences in Biotypes BIII and BIV. Cannon bone perimeter and rump height were higher in Biotype BIII. Finally, body weight was lower in Biotype BI, followed by BII, while Biotypes BIII and BIV had higher weights. Overall, the cows showed significant morphometric differences among biotypes, indicating clear structural variability. (Table 1).

The *p*-values obtained from the Shapiro–Wilk test indicate that most variables do not follow a normal distribution (*p* < 0.05), except for withers height (WH), which appears to have a normal distribution (*p* = 0.18).

**Figure 3:**
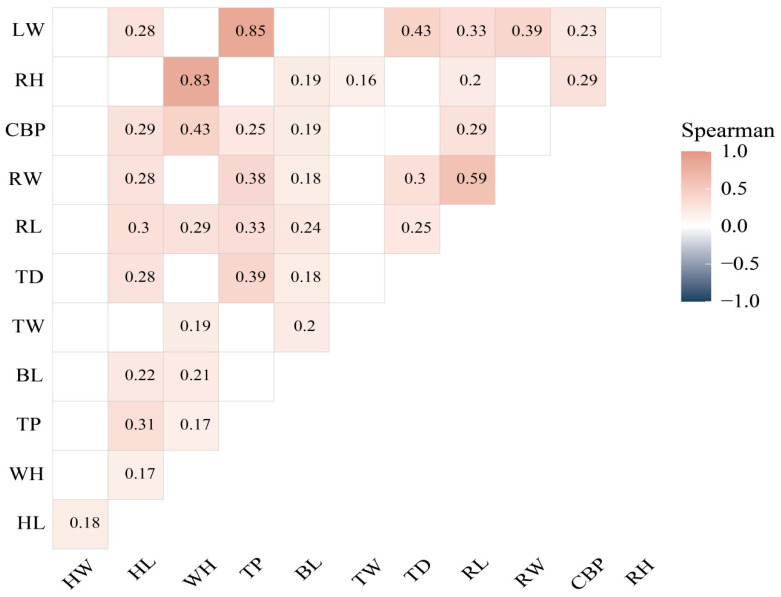
Spearman correlation between morphometric variables. Head width (HW); head length (HL); withers height (WH); thoracic perimeter (TP); body length (BL); thoracic depth (TD); rump length (RL); rump width (RW); cannon bone perimeter (CBP); rump height (RH); body weight (BW).

A Spearman correlation analysis was conducted among the morphometric parameters (Figure 3), considering only statistically significant associations. LW showed significant and positive correlations with HL, TP, TD, RL, RW, and CBP (r = 0.23–0.85). RH was significantly and positively associated with WH, BL, TW, RL, and CBP (r = 0.16–0.83). CBP showed positive correlations with HL, WH, TP, BL, and RL (r = 0.29–0.43). RW correlated positively with HL, TP, BL, TD, and RL (r = 0.18–0.59). RL showed positive correlations with HL, WH, TP, BL, and TD (r = 0.24–0.3). TD was positively associated with HL, TP, and BL, while TW correlated with WH and BL (r = 0.18–0.39). In turn, BL showed significant correlations with HL and WH (r = 0.22), and TP was also associated with these two variables (r = 0.17–0.31). Finally, a significant correlation was observed between WH and HL (r = 0.17), and the latter also correlated with HW (r = 0.18).

### 3.2. Zoometric Indices

The zoometric indices of the high-Andean Creole cows showed significant differences among the four biotypes analyzed. The anamorphosis index was highest in Biotype I compared to other biotypes, while the body index was highest in Biotype II compared to the other biotypes. In contrast, the pelvic index showed no significant variation among the biotypes. The proportionality index showed higher values in Biotype III, followed by Biotypes BII, BIV, and BI. The cephalic index was higher in Biotypes BI, BII, and BIV compared to Biotype BIII. The dactyl-thoracic index was significantly higher in Biotype III with Biotypes BI, BII, and BIV. Additionally, the transversal pelvic index and the longitudinal pelvic index were highest in Biotypes III and IV compared to Biotypes BII and BI. No significant differences were found in the pelvic index and the cephalic index among the biotypes (Table 2).

The *p*-values obtained from the Shapiro–Wilk test indicate that most variables do not follow a normal distribution (*p* < 0.05), except for Longit (*p* = 0.33), which appears to follow a normal distribution.

**Figure 4:**
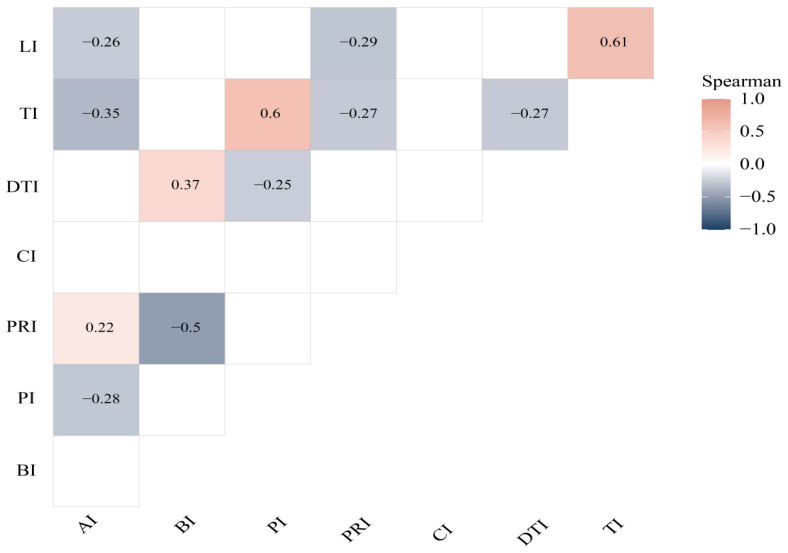
Spearman correlation between zoometrics variables. Anamorphosis index (AI); body index (BI); pelvic index (PI); proportionality index (PRI); cephalic index (CI); dactyl-thoracic index (DTI); transversal pelvic index (TI); longitudinal pelvic index (LI).

A Spearman correlation analysis was conducted among the zoometric indices (Figure 4), considering only statistically significant associations. AI showed negative correlations with PI, TI, and LI (r = 0.28–0.35) and exhibited a positive correlation with PRI (r = 0.22). BI exhibited a positive correlation with the DTI (r = 0.37) and exhibited a negative correlation with PRI (r = 0.5). In turn, PI was positively correlated with TI (r = 0.6) and negatively correlated with the DTI (r = 0.25). DTI also showed a negative correlation with TI (r = 0.27). Finally, TI was positively correlated with LI (r = 0.61).

**Figure 5:**
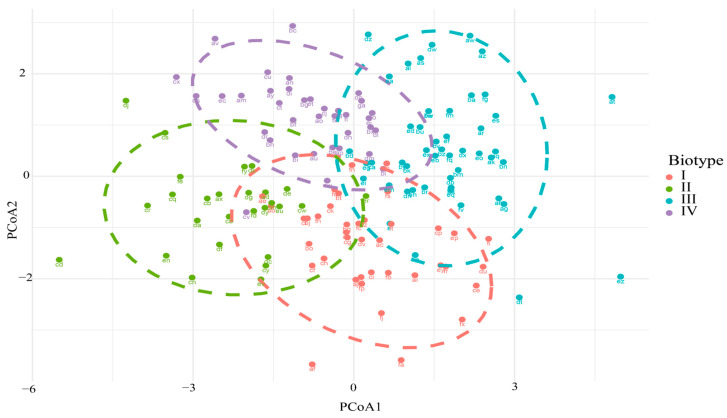
Principal Coordinate Analysis (PCoA) based on the Bray–Curtis distance between biotypes.

A Principal Coordinates Analysis (PCoA) was performed using Bray–Curtis distance to assess dissimilarity between individuals according to their biotype. Figure 5 shows the distribution of individuals in the two-dimensional space defined by the first two PCoA axes, grouped into four differentiated biotypes, which were delineated through a prior clustering analysis. This visualization revealed some degree of separation between the biotypes, with overlapping areas among certain groups. The validity of this clustering was statistically supported by a permutational analysis of variance (PERMANOVA), which revealed significant differences between biotypes (*p* = 0.0001) (Table 3).

## 4. Discussion

In this study, four biotypes of Creole cows were identified based on the analysis of morphometric and qualitative variables (Table 1, Figure 2). The Principal Coordinates Analysis (PCoA), supported by the PERMANOVA test, confirmed the morphological separation among biotypes, reflecting a structural organization consistent with patterns of adaptation and productive orientation [13]. This differentiation aligns with the morphometric measurements and zoometric indices, reinforcing the biotypological validity observed in this Creole population. The observed morphological divergence can be attributed to natural selection processes under restrictive environmental conditions, characterized by climatic variability, low forage availability, and limited access to water [14]. These factors have favored the persistence of resistant, functional phenotypes over time that have adapted to the demanding conditions of the high Andes [15,16]. Within this context, three Biotypes (I, III, and IV) were identified with a tendency toward beef production, characterized by greater body length, a wider rump, and higher body weight; and one Biotype (II) was associated with a slenderer morphology, longer head, and elongated body proportions, associated with dairy-type biotypes. This functional diversity has also been documented in other regions of Peru, such as the Amazon, where beef-oriented and dual-purpose biotypes have been reported [8].

The morphometric values provided quantitative support for the differentiation among biotypes. For example, head width was lower than that reported for Creole cattle in Ecuador and Puno [9,10], which may indicate reduced cranial robustness. The head length of cows in the four biotypes was lower than the values reported in Puno [9]. However, all showed shorter head lengths compared to cows from Ecuador [10], while Biotypes II and IV matched those found in Puno [9].

Furthermore, withers height was greater in cows of Biotypes I, II, III, and IV compared to those from Ecuador and Colombia [10,17], but lower than the values reported for biotypes from Puno [9]. Regarding the thoracic perimeter, higher values were observed compared to Ecuador [10], although they were lower than those reported for biotypes from Puno [9]. In contrast, body length was shorter than cows from Ecuador and those of Biotypes I, III, and IV from Puno [9]. These morphometric dimensions suggest elongated body structures, typical of dual-purpose biotypes.

Thoracic depth in Biotype III was similar to that reported for biotypes from Puno [9], although these were lower than the values observed in Ecuador [10]. Additionally, the values recorded for Biotypes III and IV were comparable to those of Biotypes I and II in cows from the Peruvian Amazon [8]. Rump length and width were lower than those described for cows from Puno [9] but similar to the reports from Ecuador [10]. Likewise, the measurements of Biotypes I, II, and IV were lower than those observed in cows from the Peruvian Amazon [8]. These results are considered adequate for reproductive efficiency and muscle development.

Body weight differentiated the biotypes: Biotypes I and II showed lower weights, indicating a dairy tendency, while Biotypes III and IV had higher weights, associated with a beef orientation. However, all values were higher than those reported in the Peruvian Amazon [8], possibly due to nutritional or ecological factors.

In addition to basic morphometric traits, the significant correlations among morphometric variables allowed the key functional associations to be identified between the cephalic, thoracic, and pelvic regions. HL showed positive relationships with several body parameters such as TP, TD, RL, RW, CBP, and LW. This pattern suggests that, unlike what has been reported for finer-framed, dairy-oriented animals [10], in this high-Andean Creole population, cephalic length is associated with a more robust body structure, which is characteristic of mixed or beef-type biotypes. TP showed a positive correlation with LW, reinforcing its value as a reliable predictor of body mass, which is in line with findings from studies on Creole cattle in Puno [9]. Likewise, variables such as rump length (RL), rump width (RW), and rump height (RH) also showed positive correlations among themselves and with other major body dimensions supporting their relevance in determining both body weight and reproductive and productive efficiency. These morphometric associations support the functional integration between body regions, reflecting biotypes with anatomical structures consistent with their productive orientation [9,18]. In general, the heavier biotypes showed more pronounced development along the thoracic axis, associated with greater respiratory capacity and muscle mass support. In contrast, biotypes with more developed cephalic regions but lower body mass exhibited finer body conformation, which is linked to a dairy tendency. This anatomical balance, suggesting structural compensation among regions, is consistent with empirical selection processes and long-term adaptation to the extreme conditions of the high-Andean ecosystem [19].

The zoometric indices complemented the morphometric findings by providing additional functional information. The AI was consistent with a dairy aptitude across all four biotypes, which is in agreement with studies conducted in Puno [9]. The body index (BI) indicated that all biotypes were brevilinear, a trait associated with hardiness, resilience, and adaptability to rugged Andean terrain, similar to findings reported in Puno [9].

The PI was similar across all biotypes, indicating pelvises that are wider than they are long—a condition that favors the ease of calving—as a wider pelvis facilitates the passage of the calf during parturition [20]. Furthermore, the values obtained were higher than those reported in cows from Puno [9], and similar to those found in cows from Ecuador [10]. Regarding the PRI, it reflected a rectangular body conformation, which is a common trait in beef-type animals, particularly in Biotypes III and IV [20].

The cephalic index (CI) in the four biotypes indicated a dolichocephalic morphology, characterized by a narrow head [20]. These values were lower than those observed in Creole cattle from Puno and Ecuador [10], suggesting a greater prevalence of this cranial trait in the evaluated biotypes.

Regarding the DTI, Biotype IV exhibited the lowest value below <10, indicating a finer bone structure typically associated with dairy-type animals. In contrast, Biotypes I, II, and III showed values equal to or greater than >10, reflecting greater bone mass and a more robust conformation, which are features commonly linked to beef-oriented phenotypes [20]. These findings are consistent with previous reports from Ecuadorian Creole cattle [10], where the Negro Biotype had a DTI of 9.9, while the other biotype (Encerado, colordo, and Cajamarca) showed values equal than 10 suggesting the coexistence of biotypes with different productive tendencies across regions. Similarly, these values align with those reported in Creole cows from Puno, which averaged a DTI of 10 [9].

Likewise, the TI supports this functional differentiation. Biotypes I, III, and IV recorded values equal to or greater than 33, classifying them as beef-type animals. In contrast, Biotype II, with a value below 33, was categorized within the dairy group [20,21]. However, these values were lower than those reported in Creole cows from Ecuador and Puno [9,10].

Finally, the LI supported the observed functional categorization. Biotypes I, III, and IV exhibited body proportions consistent with a beef production orientation, whereas Biotype II displayed a more elongated conformation, typical of animals specializing in dairy production [20,21]. It is worth noting that the LI values recorded were lower than those reported in Creole cows from Ecuador and Puno [9,10], which may reflect morphological differences related to genetic, nutritional, and environmental factors specific to each region [8].

The correlations among zoometric indices also provided insights into the structural coherence of the biotypes. The AI showed negative correlations with the PI, the TI, and the LI, indicating that a greater relative respiratory capacity is associated with more slender and less laterally expanded structures, which aligns with dairy-type biotypes [9,10]. The BI was positively correlated with the DTI, suggesting that animals also exhibit greater bone robustness, which is a trait typical of beef-oriented biotypes [9,10]. Likewise, the positive relationship between PI and TI reinforces the association between a wide pelvis and greater transverse development, while the positive correlation between TI and LI indicates body proportionality, which is typical of dual-purpose animals, as documented in Creole cattle from Amazonas and Puno [8,9].

## 5. Conclusions

Four biotypes of high-Andean Creole cattle were identified, each exhibiting significant differences in their morphometric traits and zoometric indices. Biotype I demonstrated notable structural robustness and pronounced thoracic development, which are traits associated with an intermediate aptitude for beef production. Biotype II displayed more refined proportions, increased cranial length, and lower body mass, suggesting a phenotype oriented toward dairy production. Biotype III combined well-developed muscle mass, pelvic width, and thoracic depth, corresponding to a beef-oriented profile, complemented by adequate reproductive functionality. Meanwhile, Biotype IV, despite showing a dactyl-thoracic index indicative of a dairy tendency, was characterized by a rectangular body conformation, higher live weight, and a broad pelvis—traits that reinforce its potential as a dual-purpose biotype (meat and milk), with favorable calving ease. This study enabled the characterization of Creole cattle biotypes in the Southern Andean region with differentiating productive orientations: beef, dairy, and dual-purpose cattle. These distinctions reflect not only the selective pressure exerted by the challenging high-Andean environment but also the influence of local empirical management practices, which have favored certain phenotypic traits according to production needs. The observed functional diversity highlights the value of high-Andean Creole cattle as a highly adapted genetic resource for marginal environments. It provides a solid technical foundation for the development of strategies aimed at conservation, genetic improvement, and sustainable utilization in extensive production systems. In this context, recognizing and harnessing this phenotypic variability is key to strengthening food security and enhancing the resilience of livestock production in high-altitude Andean regions.

## Figures and Tables

**Table 1:** Morphometric measurements (means ± SEM) of biotypes.

Variables	BI	BII	BIII	BIV
HW	19.34 ± 0.19 **^b^**	19.74 ± 0.22 **^b^**	19.67 ± 0.26 **^b^**	20.5 ± 0.22 **^a^**
HL	46.57 ± 0.35 **^b^**	47.34 ± 0.39 **^b^**	49.78 ± 0.46 **^a^**	50.19 ± 0.40 **^a^**
WH	112.13 ± 0.45 **^d^**	119 ± 0.96 **^b^**	122.11 ± 0.7 **^a^**	115.25 ± 0.6 **^c^**
TP	156.35 ± 0.7 **^b^**	155.68 ± 0.8 **^b^**	163.67 ± 1.0 **^a^**	163.86 ± 0.86 **^a^**
BL	130.0 ± 0.7 **^c^**	134.66 ± 0.9 **^ab^**	135.26 ± 1.0 **^a^**	132.33 ± 0.9 **^bc^**
TD	61.59 ± 0.6 **^bc^**	61.52 ± 0.8 **^c^**	68.29 ± 0.9 **^a^**	65.25 ± 0.8 **^b^**
RL	39.89 ± 0.4 **^d^**	41.53 ± 0.5 **^c^**	4507 ± 0.6 **^a^**	43.17 ± 0.5 **^b^**
RW	37.3 ± 0.6 **^b^**	38.21 ± 0.7 **^b^**	42.04 ± 0.8 **^a^**	41.33 ± 0.7 **^a^**
CBP	15.75 ± 0.2 **^c^**	16.16 ± 0.1 **^b^**	17.93 ± 0.2 **^a^**	16.22 ± 0.1 **^b^**
RH	114.37 ± 0.6 **^c^**	125.82 ± 0.7 **^a^**	124.37 ± 0.8 **^a^**	118.08 ± 0.7 **^b^**
BW	308.78 ± 6.52 **^a^**	303.34 ± 7.4 **^a^**	359.41 ± 8.79 **^b^**	362.11 ± 7.6 **^b^**

Head width (HW); head length (HL); withers height (WH); thoracic perimeter (TP); body length (BL); thoracic depth (TD); rump length (RL); rump width (RW); cannon bone perimeter (CBP); rump height (RH); body weight (BW); standard error of the mean (SEM). Morphometric measurements were recorded in centimeters. **a**-**b**-**c**-**d** indicate significant differences (*p* < 0.05) among biotypes.

**Table 2:** Zoometric indices (means ± SEM) of biotypes.

Variables	BI	BII	BIII	BIV
AI	2.99 ± 0.1 ^a^	3.23 ± 0.07 ^b^	2.68 ± 0.08 ^c^	2.73 ± 0.07 ^c^
BI	83.34 ± 0.7 ^b^	86.58 ± 0.75 ^a^	82.72 ± 0.89 ^bc^	80.87 ± 0.77 ^c^
PI	93. 57 ± 1.3 ^a^	92.17 ± 1.49 ^a^	93.29 ± 1.77 ^a^	96.03 ± 1.53 ^a^
PRI	86.51 ± 0.6 ^c^	89.23 ± 0.8 ^ab^	90.2 ± 0.9 ^a^	87.21 ± 0.8 ^bc^
CI	41.63 ± 0.5 ^a^	41.77 ± 0.6 ^a^	39.72 ± 0.7 ^b^	40.86 ± 0.6 ^ab^
DTI	10.08 ± 0.09 ^c^	10.39 ± 0.1 ^b^	10.96 ± 0.1 ^a^	9.9 ± 0.1 ^c^
TI	33.14 ± 0.5 ^bc^	31.92 ± 0.6 ^c^	34.44 ± 0.7 ^ab^	35.91 ± 0.6 ^a^
LI	35.5 ± 0.4 ^b^	34.69 ± 0.5 ^b^	36.92 ± 0.5 ^a^	37.49 ± 0.5 ^a^

Anamorphosis index (AI); body index (BI); pelvic index (PI); proportionality index (PRI); cephalic index (CI); dactyl-thoracic index (DTI); transversal pelvic index (TI); longitudinal pelvic index (LI); standard error of the mean (SEM). Zoometric indices were calculated based on morphometric measurements in centimeters. a-b-c indicate significant differences (*p* < 0.05) among biotypes.

**Table 3:** PERMANOVA analysis between biotypes of Creole cows.

	Df	SumOfSqs	R^2^	F	*p*
Biotype	3	520.87	0.2895	19.962	0.0001 ***
Residual	147	1278.59	0.711		
Total	150	1799.47	1		

*** *p* < 0.001 (highly significant).

## Data Availability

No new data were created or analyzed in this study. Data sharing is not applicable to this article.

## Supplementary Materials

The following supporting information can be downloaded at https://www.mdpi.com/article/10.3390/vetsci12080782/s1. Figure S1. Coat colors of high Andean Creole cows in southern Peru. (A,B) black; (C) reddish bay; (D) dark brown; (E) light brown; (F) Black overo “callejón”; (G) “Berrendo/Mora Negra”; (H) smoky/Cardeno; (I) brindle “Atigrado”; and (J) mulatto.
