# Supplementary material for: Morphometric Characterization and Zoometric Indices of High-Andean Creole Cows from Southern Peru
Source: Vet Sci. 2025 Aug 20;12(8):782. doi: 10.3390/vetsci12080782 (PMC12390703; doi:10.3390/vetsci12080782)
Supplement: Supplementary file 1 [file vetsci-12-00782-s001.zip › vetsci-3670317-supplementary.pdf]

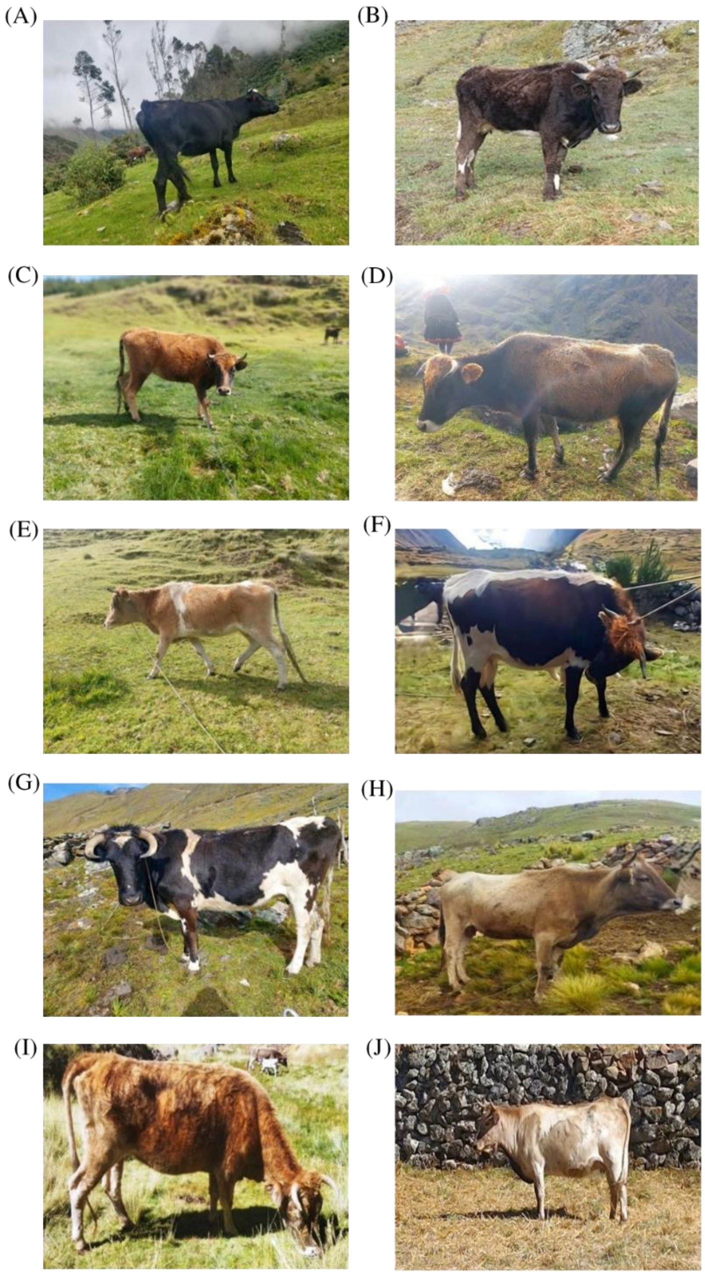

Figure Supplementary S1. Coat colors of high Andean Creole cows in southern Peru. A and B) black; C) reddish bay; D) dark brown; E) light brown; F) Black overo "callejón"; G) "Berrendo/ Mora Negra"; H) smoky / Cardeno ; I) brindle” Atigrado” ; and J) mulatto
